# Supplementary material for: Prevalence of HPV genotypes and assessment of their clinical relevance in laryngeal squamous cell carcinoma in a northeastern state of Brazil—a retrospective study
Source: PeerJ. 2022 Jul 12;10:e13684. doi: 10.7717/peerj.13684 (PMC9285469; doi:10.7717/peerj.13684)
Supplement: Supplemental Information 1 [file peerj-10-13684-s001.docx]

**>11L AACCAATAATATTGTCGTTTGTGCTGCGGGTTTACTTCTGGTACTACCAATAAGACACTAACTATAGAAGAGAAGTAGAAAAGAAAGGGAGGAATATGAACTACAGTTTATTTATCAAACCCCTTAAAAAGG**

**>14L ACTGCTGGGGCACCCCCCAACAGAGGTCTCTGTACGGAGATCTCTTAGAACTTACATAAGATACTAACTTTAAGGAGTAGCTTTTAAATGGGGGGGAATATGATTTACAGTTTATTTTTCAACCCTTTTTGGG**

**>15L no detection**

**>18L**

**GGGGTGGGGGGGGGGAAAAAGATGGCATTATGTGCTGCCTATCTACTTAGAACCTACATATAAAAATACTAACTTTAAAGAGTACCTACGACATAGGGGAGGAATATGATTTACAGTTTATTTTTCAAGCCTTAGGG**

**>22L**

**CTGTAAGCGATGGCAAGGAATTGTCTTTGTGCGGCCGATCTCTTCTAACTACTATAAAAATACTAACTTTAAGGAGTACCTAAAAATGGGGGGGAATATGATTTACAGTTTATTTTTCAAACTTTTGGG**

**>23L ATTTTCTCTCCGCCCCAAATGTCCTATGTGCTGCCATATCTACTTCGGAACTTATATAAAAATACTAACTTTAAGGAGTACCTATGACATGGGGAGGAATATGATTTACAGTTTATTTTTCAACCCCTTTAAGG**

**>24L ATCACCGGGTGGCCCCCGGAACGATGTCTTATGTGCTGCCATATCTACTTCAGAAACTACATATAAAAATACTAACTTTAAGGAGTACCTACGTTATGGGGAGGAATATGATTTACAGTTTATTTTTCAA**

**>30L CCTCCGTACGCCCCCAAGACGTTGTCTTATGTGCTGCATATCTACTTCGAAACTACATATAAAAAATACTAACTTTAAGGAGTACCCACTATATGGGGAGGAATATGATTTACAGTTTATTTTTCAACCCTTTAA**

**>31L TTTTTTATGTAAAAACGAAAAACAGCCTCAAAATGCAATCTACTTCTGAAACTACCAGAAGAATACTAGCTTTAAGGAGTTCCCCCCTTTTTGGGGGGGAGTATGATTTACAGTTTATTTTTCAAACCTT**

**>32L**

**GCAAAAAAATTGGTCTTTAGGTGCTGGCATATCTTCTTGAGAGCCTACATATAAAAATACTAACTTTAAAGAGTACCTACGAAATGGGGAGGAATATGATTTACAGTTTATTTTTCAACCCCTTAAG**

**>33L GGCTTCGGTGTGGCCCACGAATTGTCTCTGTGATGCATATCTCTTCTAAACTACTATAAAATACTAACTTTAACGAGTACCTATCATTTGGGGGGGAATATGATTTACAGTTTATTTTTCAAACCCTT**

**>34L**

**CCCCCCCTTTTTTTTTTGTTGGGGGGGGGGGGGAAAAAAAAAACCCCCCCCGAG**

**>35L CCGGTCCGGTGGGGCCAAACCGTGGCCTATGTGCGGCATATCTCTTCGAACTACTATAAAAATACTAACTTTAACGAGTACCTATGACATGGGGGGGAATATGATTTACAGTTTATTTTTCAAACCTTTTTT**

**>36L CAAAAAAATGGTCATTAATTGTGCTGCCATATCTACTTCAGAAACTACATATAAAAATACTAACTTTAAGGAGTACCTATTTAAATGGGGAGGAATATGATTTACAGTTTATTTTTCAAGCCCTTTTAAAAA**

**>37L AGGGGGGGGCGGGGGACCAGTGCTTTTGTGGGCATATCTCTTAGAAACTTCTATAAAAATACTTGGTTTAAGGAGTACCCCTTACATGGGGGGGAGTATGATTTACAGTTTATTTTTCAAGCCCTTTAGG**

**>38L GTAGTGCCTGAGGGGCCCCCGGGGAGCCTCCTGGGGTGCATAGCTTCTTTTGTACTATTGGAAGAGACTTACTTTAAGGAGTAGCTAAAAATGGGGGGGAGTATGATTTACAGTTTATTTTTCAACCCTTTTAG**

**>39L TTTTGAATGCTGAGCGGGCAAAGGTCTAGTGCTGCCTATCTACTTAGAAACTACATAGAAAAATACTAGGTTTAAGGAGTACCCATGATTTGGGGGGGAATATGATTTACAGTTTATTTTTCAACCTT**

**>40L GTACCCGCCCGGCCCCGGCAAAGGCTCCGTGAGGCATTCTACTTATAAACTTATATAAAAATACTAACTTTAAGGAATACCCACTTTATGGGGGGGAATATGATTTACAGTTTATTTTTCAACCTT**

**>42L TAAAAGATGGTCATTATGTGCTGCCATATCTACTTCAGAACTACATATAAAAATACTAACTTTAAGGAGTACCTCCGACATGGGGAGGAATATGATTTACAGTTTATTTTTCAACCCTTTAGG**

**>43L GCTCGCATGCCCCCCAAACAAATGTCATTATGTGCTGCCTATCTACTTCAGAACTACATATAAAAATACTAACTTTAAAGAGTACTAAACAGGAAGGGAGGAATATGATTTACAGTTTATTTTTCAAAGAAAAAAAAAAAGGCC**

**>44L GTTTAGCTCTGGTACGGGGCATAAAGTATATGGCTGTCAGATCTATTTCTGAGACTACATATAAAAATACTAAGTTTAAGGAGTAGATACTTAAGGGGGGGAATATGATTTACAGTTTATTTTTCAACCCTTTTAGTGGGGCCTTGTGGTCGTGCAGTATGTCCTCAATAGTTTGGATTTTGTTATTGCCCTACTCAGAGGGAGTAAATATAACTCCACGAAAATCAGCATCTATGTCCGGTTGTGCCGTGCGCGGTGGGGCAGGTTTTTATCTCCTTTATTTTTTTTAATTCTTTATTGCCTATGGGAGGGGGCCTGCCCCCCCCCCCCCCCCCCCCCCCCCCCCCCCCCCCCGCCCCTAACATATATGGTGTCGGATTGGCGGAATAGACCCTTATGTG ACAGCGCACGCTTAATCCCCCCCCGCCCACAACCCCCCCGTAAATCATGTGTGTGGCTGAACCTGTAGGATCGTGTGTACCCCCCCCTTTTAATTTTTTTTCCTGAGTTTATAGGGGAAAAAAACGAATAAAACAAATAAATG**

**>48L GATTTTGTTGCACGCAAAAATGTCTCTTTGATCATATTTACTTTTGACGACTAGAAAATACTAAGCAAAAAAGACCTCTTGTTGGGGAGGAATATGATTTACAGTTTATTTAACAAACCCTTTAG**

**>49L**

**GTGAGGAGTGGGCCGAAAAGTGTATATGTGCTTTATTGTCTTCGAACTGGGAAAAGAACTTACGTTAAGGAGACCTATTATTTGGGGGGGAGGTGATTTACTTTGATTTTTCAACAACTTTAAG**

**>50L ACGCCCGGAAATAGGTCTTATGTTGCTGCATATCTACTTCTGAACTACATATAAAAATACTAACTTTAAGGAGACCTATTATATGGGGGGGAATATGATTTACCGTTTATTTTTCAACCCTTTTAAG**

**>51L AAAAAATTGGTCTTTATGTGCTGGCCATATCTACTTCAGAAACTACATATAAAAATACTAACTTTAAGGAGTACCTATTATTTGGGGAGGAATATGATTTACAGTTTATTTTTCAACCTTTTTA**

**>52L GTATATTGGGGGGGGGGCAGGACTAGTCTCTGTGCTGCGTATCTCTTCAGGTACTCCTTAATTCACAAACTAAGAAGAGTAGTTAAAGTGGGGAGGAGGATGATCTACTTTTATTTATCAACCCCTTAAC**

**>53L ACTCGTCCTATAAACGCAACCAAAAGTATTATGTGCTGCATATCTACTTCTAAACTACATATAAAAATACTAACTTTAAGGAGTACCCATTATTTGGGGAGGAATATGATTTACAGTTTATTTTTCAA**

**>57L TGGGTTTCCTAAAAAAACAAAACAAAGACTTTGTACATTGTATCTCTTCTGCTACCTCCACTAATTCACATATATGAATACATGTTTAAGTAAGAGGATATGATTTCA**

**>62L TGCTCTCTTTTAGGGGCACAAAAAGGTATCATGTGCTGCCATATCTACTTCAGAAACTACATATAAAAATACTAACTTTAAGGAGTACCTATGAAAATAGGGGAGGAATATGATTTACAGTTTATTTTTCAAGCCTTTAG**

**>63L TATTCTGTTATAGGGGGCACCAATTGTATTTGTGCTGCCATATCTACTTCGAAACTACATATAAAAATACTAACTTTAAGGAGTACTTGACGGGAAGAGGAATATGATTTACAG**

**>64L GAACCTTAGGCTAAACGGCCAGCAAAAGGCCTTAATAATGCATATCTACTTCTATACTACATATAAGAAACTAACTTTCACGAGTACCTCCGTTTTGGGGGGGAATATGATTTACAGTTTATTTTTCAA**

**>65L AGTTGTTCGGCGGGGCCGCAAAAGGACTATGTGGGGCATTCTCTTCTAACTTATAGAAAAATACTAGCTTTAAGGAGTACCTATTAAATGGGGGGGAATATGATTTACAGTTTATTTTTCAACCTTTAAGG**

**>66L CCTTCTGGGGCAGCGGGGCAATAGGATCTGTGCGGATATCTACTTCTGAACTACATAGAAGATACAAGCTTTAAGGAGTACCCCCTTTATGGGGGGGAATATGATTTACAGTTTATTTTTCAACCCTTA**

**>67L no detection**

**>68L no detection**

**>69L CGTTTGGATTTGGCGTTGTGTGGTGGTCTATCTACTTCAGGGACTACATATAAAAATACTAACTTTAAGGAGTACCTACGTAAATAAGGGGGGAATATGATTTACAGTTTATTTTTCAAACCCTTTTAGGGG**

**>71L TCCGTTTTAATTGAACTTTATGGACACAAGTAACTAGTGACTGTACATGTAAAAAGGAAAATTGTGAAGAATATATAAGAAAGATGGAGAATATGATTTACAGTTTATTTTTCAAACATGTCTTATAAATTCTTTAAAATTTTCATTTTTATATGTACTGTCACTAGTTACTTGTGTGCATAAAGTCATATTAGTACTGCGAGTAGTATCTACCACAGTAACAAAA**

**>72L CGGTTTAATTTGTTCTTATGTGCTGCATATTTACTTCATAAACTACATATAAAAATACTAACTTTAAGGAGTACCTATTCACATGGGGGGGAATATGATTTACAGTTTATTTTTCAATCCCCTTTAAGG**

**>73L CGGTCCCATTGGTCCTTTATGTGCTGGCCTATCTACTTTATAAACTACATATAAAAATACTAACTTTAAGGAGTACCTATGACATGGGGGGGAATATGATTTACAGTTTATTTTTCAAACCCTTTTAA**

**>75L AAAAAAATGTTATATATGTGGCTGCATATCTACTTCTGAAACTACATATAAAAATACTAACTTTAAGGAGTACCTATGAAAAAGGGGAGGAATATGATTTACAGTTTATTTTTCAACCCCTTTTAGGGG**

**>76L GGATAATGGCGCGCAGTCGAAGTCTATGTGCTGCATATTTCTTATAACTTTTATAAAAATACTAACTTTAAGGAATACCCATTACTTGGGGGGGAGTATGATTTACAGTTTATTTTTCAAACCCTTAAC**

**>79L no detection**

**>81L ACGGCCGATTGGCATTTTGGTGCTGGGATATCTACTTCAGAAACTACATATGAAAATACTAAGTTTAAGGAGTACCTATGAAATAAGGGAGGAATATGATTTACAGTTTATTTTTCAAACCCTTTTTAAGG**

**>82L AGCATTGGTTTAAAGGGAGGAAAAATGTCTGGTGTTCTGCTGTGTCTACTAGTGACAGGACATATAGAAATGACAATTTTAAGGAATATTTAAGGCATGGTGAAGAATATGATTTACAGTTTATTTTTCAAACCATGTTCTTAAATATTCCTTAAAATTGTCATTTTTATATGTACTGTCACTAGTAGACACAGCAGAACACACAGACATATTTGTACTACGGGTAGTATCTACCACGATACCAAA**

**>83L ACCGTGCTCTGCCCGCCGCGCCATAGTCTTATGTGCTGCATATCTACTTATAAACTACTATAAAAATACTAAGTTTAAGGATACCTATTACTTGGGGGGGAATATGATTTACAGTTTATTTTTCAAGCCTTAAG**

**>88L ATTACTCCTCATATCACACAAACAGCCCCTATGTGCTCTGTGTCTATTCTGCTACATCCACTAATTCACATTAAATGAATACATGCTTTAAAATAAAAGGGAATATGATTTACAATTTATTTTTCATCAACTCCTTTAAAAGA**

**>89L CTCCTTCCCCTCTTGCCAGATGATTGCCCTTGTGCTTGTGTCTATTCTGCTTCATCCCTAATTCTTTTTCCTTGAATACTTGCTTAAAAAGGGGGGATATGATTTTCCGTTTATTTTTCACACCCCCTTTTTATGGTC**

**>90L ACAAAATTGACCTTCCGTGCTGCAGTATCTACTTCTGCTACTTCCTCTAAGTCACAAACAAGGAAGAGAAGTATAAGAAGGAGCAGGATTGATTTACTACAGTTTATTCATCAACCCTTTAATG**

**>93L no detection**

**>95L TGGGTGTGTTGTGGCGCAGCCAATGACCTCGTGCCTCGGGGTTAATTCTGCTCTTCCTCTAATTCACATTCGCTTGAAGAGATGCTTTAGGAAGGGGGGAGTATGATTTACAATTTATTTTTCA**

**>104L GAAAAAAAGGGTATTATTGTGCTTGCCATATCTACTTTCAGAAACTACATATAAAAATACTAACTTTAAGGAGTACCTATGACAAAGAAGGAGGAATATGATTTACAGTTTATTTTTCAACTTCTTTAAAAGG**
